# Racial, socioeconomic, and neighborhood characteristics in relation to COVID-19 severity of illness for adolescents and young adults

**DOI:** 10.1093/pnasnexus/pgad396

**Published:** 2023-11-17

**Authors:** Ayaat Dahleh, Andrew J Bean, Tricia J Johnson

**Affiliations:** The Graduate College, Rush University, Chicago, IL 60612, USA; The Graduate College, Rush University, Chicago, IL 60612, USA; Department of Health Systems Management, Rush University College of Health Sciences, Chicago, IL 60612, USA

**Keywords:** COVID-19, hospitalizations, adolescents, young adults, youth, public health, social determinants of health

## Abstract

This study tests the hypotheses that insurance status, race and ethnicity, and neighborhood characteristics are associated with hospital admission and severe health outcomes (Intensive Care Unit [ICU] admission and oxygen assistance) for youth and young adults who present to the emergency department (ED) with COVID-19 in a single, academic health system in Illinois, Rush University System for Health (RUSH). Demographic and clinical data from the electronic health record were collected for all 13- to 24-y-old patients seen at RUSH who tested positive for COVID-19 between March 2020 and 2021. Individual-level and neighborhood characteristics were analyzed to determine their association with hospital admission and severe health outcomes through generalized estimating equations. As of March 2021, 1,057 patients were seen in the ED within RUSH in which non-Hispanic White (odds ratio [OR], 2.96; 95% CI, 1.61–5.46; *P* = 0.001) and Hispanic (OR, 3.34; 95% CI, 1.84–6.10; *P* < 0.001) adolescents and youth were more likely to be admitted to the hospital compared with non-Hispanic Black/other adolescents and youth. Patients with public insurance or who were uninsured were less likely to be admitted to the ICU compared with those with private insurance (OR, 0.24; 95% CI, 0.09–0.64; *P* = 0.004). None of the neighborhood characteristics were significantly associated with hospital admission or severe health outcomes after adjusting for covariates. Our findings demonstrated that race and ethnicity were related to hospitalization, while insurance was associated with presentation severity due to COVID-19 for adolescents and young adults. These findings can aid public health investigators in understanding COVID-19 disparities among adolescents and young adults.

**Significance Statement**

Assessing current youth and young adults’ social risk factors for COVID-19 health outcomes is crucial for determining immediate and long-term impacts of the pandemic on this vulnerable population. The findings may encourage policy creation toward population health-related services nationally and globally to see a decline in COVID-19 severity among underserved youth.

As of December 2022, the severe acute respiratory syndrome disease (COVID-19) caused by coronavirus 2 (SARS-CoV-2) had impacted >625 million individuals worldwide with over 6.5 million deaths (1). Between March 2020 and August 2021, the incidence for adolescents aged 12–17 was 63.7 per 100,000 individuals, and the cumulative incidence of hospitalizations related to COVID-19 for children and adolescents was 49.7 cases per 100,000 individuals in the United States (2). However, findings regarding adolescent and youth predisposition to COVID-19 compared with adults are limited and inconsistent. For example, some studies have demonstrated that adolescents and youth are much less susceptible to COVID-19 compared with older adults (3–6). Other findings have shown that the incidence and severity of COVID-19 among youth and adolescents was, in fact, greater or comparable with that of adults (7–11). The reported risk of infection by the virus may seem lower in children and youth than in adults not due to explicit differences in immune status, but rather due to a decreased likelihood of being tested for the virus, having increased preventive measures, and having a higher probability of experiencing asymptomatic COVID-19 outcomes compared with adults (12–14). Because outcomes regarding the risk of infection for children and youth compared with adults have remained mixed and inconsistent, assessing adolescent and youth susceptibility to COVID-19 may be crucial when examining the risk of infection, illness severity, and the formation of prevention services.

Social determinants of health (SDOHs) refer to the nonmedical conditions that an individual is exposed to according to their place of birth, residence, work, and worship (15). Understanding how SDOHs are associated with healthcare utilization, specifically related to COVID-19, is important because youth and young adult populations may be at a high risk for increased hospital use and emergency department (ED) visits due to economic factors and their vulnerable transition to adulthood without a complete understanding of how to access or utilize healthcare. Some reports have demonstrated that race and ethnicity (16–19), primary payer (20–22), and neighborhood characteristics (16, 18, 23–25) are all associated with the risk of COVID-19, although other studies have demonstrated that race and ethnicity (26–30), primary payer (30, 31), and neighborhood characteristics (17, 24, 25) are not associated with COVID-19 outcomes. Thus, while risk factors have been assessed among communities and families, the discrepancy in the above findings makes it essential to examine the association of such characteristics on youth and young adult COVID-19 outcomes.

We hypothesized that having public insurance or a lack of health insurance (low socioeconomic status), racial and ethnic minority status, and neighborhood census-tract characteristics relating to reduced income, decreased educational attainment, difficult housing conditions, and greater food insecurity are all associated with increased hospital utilization and more severe health outcomes for youth and young adults with COVID-19. While data related to disparities in COVID-19 health outcomes have been assessed among adult cohorts, an analysis of these inequalities in adolescents and youth populations is limited. Determining whether gaps exist related to COVID-19 outcomes in youth who are especially vulnerable to the consequences of unmet healthcare needs by investigating health outcomes and healthcare utilization at an academic health center, Rush University System for Health (RUSH), is important in aiding public health investigators to understand COVID-19 disparities, accurately evaluate adolescents and youth with COVID-19, and develop appropriate disease prevention strategies.

## Methods

### Ethics review

This study protocol was approved as exempt by the Rush University Medical Center (RUMC) Institutional Review Board.

### Study setting

The RUSH is an urban Midwest academic health system comprised of three hospitals: an urban academic center (RUMC), a suburban hospital (Rush Copley Medical Center), and a suburban/rural hospital (Rush Oak Park Hospital) (32). It also includes outpatient care facilities and providers that have a common electronic medical record (EMR) system (Epic) (32). Of the individuals who present to the ED at RUMC, approximately 19% are between the ages of 0 and 17, 43% are between the ages of 18 and 39, and roughly 38% are 40 years of age or older (33). Rush Oak Park Hospital presents a similar age demographic with regard to ED patients, as approximately 21% of patients are between the ages of 0 and 17, 39% are between the ages of 18 and 39, and 40% are 40 years of age or older (33). Furthermore, the community served by Rush Copley constitutes about 28% of individuals between the ages of 0 and 17, 22% between the ages of 18 and 34, and about 50% of patients are 35 years old or older (34).

### Data sources

Encounter-level data for patients who presented to RUSH with COVID-19 between 2020 March 1 and 2021 March 31 were extracted from the organization's clinical data warehouses containing Epic EMR data. Data regarding neighborhood characteristics by census tract were extracted from the American Community Survey conducted by the United States Census Bureau.

### Inclusion and exclusion criteria

Patients who presented to RUSH between March 2020 and March 2021 were included in the study if they (i) were admitted to the ED with a diagnosis of COVID-19, and (ii) were in their late adolescence or youth (13–24 years of age) at the time of their ED visit consistent with the World Health Organization's definition of youth (35).

### Variables

#### Individual characteristics

Individual-level variables included in this study were patient socioeconomic characteristics and comorbidities. The socioeconomic characteristics included the source of insurance (private, public/uninsured [including Tricare]) and race and ethnicity (Hispanic, non-Hispanic White youth, and non-Hispanic Black/other youth). Race and ethnicity were grouped into three categories to prevent small numbers in specific subgroups that would not be appropriate for meaningful analyses. Covariates included sex (male, female), age, month, and year of initial ED visit (March–October 2020 [waves 1 and 2], November 2020–March 2021 [wave 3 onward]) (36), hospital visited (RUMC, Rush Copley Medical Center/Rush Oak Park Hospital), and reported comorbidities (0–1 comorbidities, 2 or more comorbidities). Although hospitalizations from three hospitals were included, there were very few hospitalizations to one of the hospitals, and as such, we combined the two suburban community hospitals. Secondary ICD-10-CM diagnosis codes were used to determine the presence of comorbidities at the time of ED presentation using the Clinical Classifications Software Refined (37). Comorbidities included conditions previously explored in other adolescent and adult studies such as kidney disease, obesity, sickle cell, diabetes, asthma, cerebrovascular disease, hypertension, HIV, neurologic conditions, liver disease, tobacco disorder, and heart diseases (18, 23, 38).

#### Neighborhood-level characteristics

Neighborhood characteristics previously shown to be related to COVID-19 outcomes were investigated in this study (16–18, 23, 39, 40). Each patient's home address was geocoded and linked to neighborhood characteristics from the United States Census Bureau at the census tract level. Pearson Correlation coefficients were used to evaluate the correlation among neighborhood variables to ensure the uniqueness of all variables. Variables with correlations <0.7 were incorporated into the generalized estimating equation (GEE) models and included the following: percentage of the households below the poverty status in the past 12 months, percentage of the population between 18 and 24 years of age that are less than a high school graduate, percentage of the population 25 and over with <9th grade educational attainment, percentage of the population 25 years and over that were enrolled in high school but never received a diploma, percentage of occupied housing units with 1.51 or more occupants per room, unemployment rate for population 16 years and over, and percentage of households with a male being the head of the household with no spouse present.

#### Healthcare utilization outcomes

Three primary healthcare utilization outcomes were investigated. The first outcome was hospital admission, defined as either being admitted to the hospital from the ED or not being admitted to the hospital and discharged from the ED. For patients with more than one ED visit with a COVID-19 diagnosis, the first visit was included in the analysis. The second outcome was intensive care unit (ICU) admission for patients who were admitted to the hospital, consistent with previous literature that has utilized ICU admission as a measure of severity (admitted or not admitted) (18, 23, 38). The third outcome was oxygen utilization for patients admitted into the hospital. Consistent with the World Health Organization's Ordinal Scale for Improvement, a patient's state can be considered “mild disease” if they are hospitalized with no oxygen therapy (score 3) or receive oxygen administered by mask or nasal prongs (score 4) and are considered as having “severe disease” if they are given noninvasive ventilation and high-flow oxygen (score 5), or mechanical ventilation and intubation (score 6) (41). This study compared hospitalized patients who received no oxygen (score 3) vs. those who received any oxygen (≥score 4).

### Statistical analyses

#### Power analysis

An a priori power analysis was conducted using G* Power Version 3.1.9.6 for sample size estimation based on GEE modeling. With a significance criterion of alpha = 0.05 and power = 0.80, a sample size of 600 was calculated to result in an odds ratio (OR) of 1.33. Thus, our obtained sample size of *n* = 1,057 youth and young adults was sufficient to ensure appropriate testing of the study hypotheses. All remaining analyses were performed in GraphPad/Prism Version 9.2.0 and SPSS Version 26, with the significance set to *P* < 0.05.

#### Bivariate analyses

All bivariate analyses completed in this study are presented in Tables 1, 3, and 5. Chi-square tests were conducted to assess statistically significant relationships between each of the following variables in relation to each healthcare utilization outcome: sex, race and ethnicity, date of visit, hospital visited, and comorbidities. Mann–Whitney *U* tests were performed to compare the central tendencies for each of the following variables in relation to each healthcare utilization outcome: age and neighborhood characteristics. Predictive accuracy was validated through the receiver operating characteristic (ROC) curve and Pearson correlation analysis among neighborhood variables was completed to ensure the uniqueness of each variable.

**Table 1.** Description of the sample of patients by hospital admission status, *n* = 1,057.

	Total *n* = 1,057	Not hospitalized *n* = 933 (88.3%)	Hospitalized *n* = 124 (11.7%)	*P*-value
Sex, *n* (%)				0.061
Male	449 (42.5)	406 (43.5)	43 (34.7)	
Female	608 (57.5)	527 (56.5)	81 (65.3)	
Race and ethnicity, *n* (%)				<0.001
Hispanic	443 (41.9)	402 (43.1)	41 (33.0)	
Non-Hispanic Black/other	521 (49.3)	461(49.4)	60 (48.4)	
Non-Hispanic White	93 (8.8)	70 (7.5)	23 (18.5)	
Payer, *n* (%)				0.854
Private	291 (27.5)	256 (27.4)	35 (28.2)	
Public/uninsured	766 (72.5)	677 (72.6)	89 (71.8)	
Age in years, median (IQR^a^)	21 (18–23)	21 (18–23)	21 (18–23)	0.552
Date of visit				0.008
Waves 1 and 2	553 (52.2)	502 (53.9)	51 (41.1)	
Wave 3	504 (47.7)	431 (46.0)	73 (58.9)	
Hospital, *n* (%)				0.001
RUMC	479 (45.3)	406 (43.5)	73 (58.9)	
Rush Copley Medical Center and Oak Park Hospital	578 (54.7)	527 (56.5)	51 (41.1)	
Comorbidities, *n* (%)				<0.001
0–1 comorbidities	927 (87.7)	851 (91.2)	76 (61.3)	
2 or more comorbidities	130 (12.3)	82 (8.8)	48 (38.7)	
Neighborhood characteristics, median (IQR^a^)				
Percentage of households below the federal poverty line in the past 12 months	17.2 (8.8–25.4)	17.2 (8.7–25.5)	16.45 (9.3–25.3)	0.760
Percentage of the population between 18 and 24 years of age that are less than a high school graduate	14.7 (7.1–23.9)	14.8 (7–23.4)	13.95 (7.4–24.5)	0.961
Percentage of the population 25 and over with <9th grade educational attainment	6.1 (3.4–18.1)	6.1 (3.5–18.3)	6.15 (2.8–13.3)	0.171
Percentage of the population 25 years and over that were enrolled in high school but never received a diploma	10.3 (6.1–15.8)	10.3 (6.4–15.9)	10.35 (4.8–15.3)	0.194
Percentage of the occupied housing units with 1.51 or more occupants per room	0.5 (0.0–1.8)	0.5 (0.0–1.8)	0 (0.0–1.5)	0.069
Unemployment rate for population 16 years and over	7.4 (5.1–13.7)	7.4 (5.1–13.7)	7.4 (5.1–14.4)	0.771
Percentage of the households with a male head of the household with no spouse present	6.8 (3.5–9.7)	6.8 (3.5–9.6)	6.45 (3.5–10.5)	0.889

^a^IQR (25th percentile, 75th percentile).

#### Generalized estimating equations

Because it can be challenging to ensure that data are anonymized, the Health Information Portability and Accountability Act mentions identifiers that should be excluded from data to ensure that data have been properly de-identified (42). In an appropriately large cohort, such protocols make it difficult to identify patients. Yet, in a smaller data set in which data may appear aggregated, it is crucial to censor all counts smaller than a particular threshold to sufficiently mask the same aggregate patient counts. For example, the Patient Centered Outcomes Research Network utilizes a de-identification method within queries by censoring all counts smaller than a predetermined value of 11 (43). We have therefore utilized this approach in our data set and have grouped categories with small counts into larger groups and then censored aggregate counts below our threshold of <11 to protect patient privacy. This also helped in maintaining reasonable cell sizes in statistical models to ensure goodness of fit for such models.

The independent variables investigated in our models included insurance/payer status, race and ethnicity, and neighborhood characteristics to determine whether significant differences existed in hospital admission, ICU admission, and oxygen use among inpatients. The covariates that were adjusted for included sex, age, date of visit, hospital visited, and comorbidities. A GEE model with a binomial distribution for the response variable and a logit link function was utilized. The GEOIDs (numeric codes that uniquely identify the geographic areas for which the Census Bureau tabulates data) of each patient's census tract location were identified as clusters, and an independent working correlation matrix structure was used. To examine the relation of comorbidities and neighborhood characteristics on the GEE analyses, three GEE models were constructed for each healthcare utilization outcome (ED/inpatient admission, ICU admission, and oxygen use). This step-by-step modeling approach enabled us to monitor for overfitting, account for any clustering within variables, and to examine the contribution of each individual variable in relation to each healthcare utilization outcome by first analyzing the main independent variables before adding other variables such as comorbidities and neighborhood characteristics. In addition, supplemental analyses stratified by race and ethnicity for hospital admission were conducted following the same step-by-step approach (Tables S1–S3). Minimum count thresholds (>11) were utilized for each variable investigated to decrease the risk of identification from aggregate values during all GEE analyses and to ensure appropriate modeling accuracy through the elimination of small cell size counts. Variables in the models that did not exceed the minimum count threshold were either combined with other subgroups or not included in the analyses.

## Results

### ED vs. hospitalized demographics

Overall, 1,057 ED visits occurred for individuals between the ages of 13 and 24 who tested positive for COVID-19 within RUSH between March 2020 and March 2021 (Table 1). Of those 1,057 visits, 933 patients were seen only in the ED (88.3%), while 124 were admitted to the hospital (11.7%). The median patient age was 21 years (interquartile range [IQR] = 18–23). Additionally, 41.9% (*n* = 443) were Hispanic patients, 49.3% (*n* = 521) were non-Hispanic Black/other patients, and 8.8% (*n* = 93) were non-Hispanic White patients. There was no difference by insurance coverage in hospital admission. The vast majority of patients (87.7%) presented with 0 or 1 comorbidities, while 12.3% of patients presented with 2 or more. More than half (52.2%) of the ED visits occurred during waves 1 and 2. Chi-square analyses reported in Table 1 illustrated that race and ethnicity (*P* < 0.001), date of visit (*P* = 0.008), hospital visited (*P* = 0.001), and comorbidities (*P* < 0.001) were statistically associated with hospital admission, while sex, payer/insurance status, age, and neighborhood factors were not.

### Hospital admission

Table 2 reports adjusted ORs for hospitalizations vs. ED encounters after adjusting for patient socioeconomic characteristics, comorbidities, and neighborhood characteristics. Non-Hispanic White patients (OR, 2.96; 95% CI, 1.61–5.46; *P* = 0.001) and Hispanic patients (OR, 3.34; 95% CI, 1.84–6.10; *P* < 0.001) had higher odds of hospital admission compared with non-Hispanic Black/other patients. Neighborhood factors were not significantly associated with hospital admission. Source of insurance was not incorporated into the GEE models for hospital use because of low frequencies in outcome categories, which could produce unreliable model effect estimates and result in a bad model fit.

**Table 2.** GEE: associations between ED and hospital admissions in relation to independent variables, *n* = 1,057.

	Model 1: socioeconomic characteristics		Model 2: addition of comorbidities		Model 3: addition of neighborhood characteristics	
Variable^a^	Adjusted OR (95% CI)	*P*-value	Adjusted OR (95% CI)	*P*-value	Adjusted OR (95% CI)	*P*-value
*ED and inpatient admissions*						
Sex: female	1.43 (0.96–2.12)	0.077	1.71 (1.11–2.64)	0.015	1.72 (1.11–2.65)	0.015
Race and ethnicity: non-Hispanic White	2.96 (1.61–5.46)	0.001	3.50 (1.90–6.44)	<0.001	3.38 (1.72–6.64)	<0.001
Race and ethnicity: Hispanic	3.34 (1.84–6.10)	<0.001	3.56 (1.94–6.53)	<0.001	3.28 (1.71–6.30)	<0.001
Age	0.97 (0.91–1.04)	0.400	1.00 (0.93–1.07)	0.951	1.00 (0.93–1.07)	0.976
Date of visit: wave 3	1.67 (1.12–2.47)	0.011	1.61 (1.07–2.43)	0.023	1.57 (1.04–2.38)	0.031
Hospital: RUMC	2.07 (1.36–3.14)	0.001	1.71 (1.13–2.60)	0.011	1.68 (1.08–2.62)	0.022
Comorbidities: two or more			6.77 (4.24–10.80)	<0.001	7.00 (4.31–11.36)	<0.001
Percentage of the households below the federal poverty line in the past 12 months					1.00 (0.97–1.02)	0.766
Percentage of the population between 18 and 24 years of age that are less than a high school graduate					0.99 (0.97–1.01)	0.258
Percentage of the population 25 and over with <9th grade educational attainment					1.00 (0.97–1.03)	0.930
Percentage of the population 25 years and over that were enrolled in high school but never received a diploma					1.02 (0.98–1.08)	0.339
Percentage of occupied housing units with 1.51 occupants or more per room					1.03 (0.90–1.19)	0.639
Unemployment rate for population 16 years and over					1.00 (0.96–1.03)	0.876
Percentage of households with a male being the head of the household with no spouse present					1.00 (0.93–1.06)	0.888
ROC curve area	0.65		0.73		0.74	

^a^Reference groups: male, non-Hispanic Black/other, private, waves 1 and 2, Rush Copley Medical Center and Oak Park Hospital, 0–1 comorbidities.

### ICU admission demographic characteristics

Of the 124 inpatient admissions, 30.6% were admitted to the ICU (*n* = 38; Table 3). The median age of ICU patients was 19 years (IQR = 17–22). Public insurance or uninsured was most common (57.9%) among ICU patients, followed by private insurance (42.1%). More than half of the ICU patients were males (52.6%) compared with females (47.4%). Sixty-three percentage of the ICU patients (*n* = 24) presented with 2 or more comorbidities and 60.5% were admitted between March 2020 and October 2020. Chi-square analyses demonstrated that sex (*P* = 0.005), age (*P* = 0.008), insurance status (*P* = 0.022), date of visit (*P* = 0.004), and comorbidities (*P* = 0.001) were significantly associated with ICU admission, while none of the neighborhood factors were associated with ICU admission.

**Table 3.** Description of sample by ICU admission status, *n* = 124.

	Total *n* = 124	Non-ICU admission *n* = 86 (69.4%)	ICU admission *n* = 38 (30.6%)	*P*-value
Sex, *n* (%)				0.005
Male	43 (34.7)	23 (26.7)	20 (52.6)	
Female	81 (65.3)	63 (73.3)	18 (47.4)	
Payer, *n* (%)				0.022
Private	35 (28.2)	19 (22.1)	16 (42.1)	
Public/uninsured	89 (71.8)	67 (77.9)	22 (57.9)	
Age in years, median (IQR^a^)	21 (18–23)	21 (20–23)	19 (17–22)	0.008
Date of visit				0.004
Waves 1 and 2	51 (41.1)	28 (32.6)	23 (60.5)	
Wave 3	73 (58.9)	58 (67.4)	15 (39.5)	
Comorbidities, *n* (%)				<0.001
0–1 comorbidities	76 (61.3)	62 (72.1)	14 (36.7)	
2 or more comorbidities	48 (38.7)	24 (27.9)	24 (63.3)	
Neighborhood characteristics, median (IQR^a^)				
Percentage of the households below the federal poverty line in the past 12 months	16.5 (9.3–25.3)	16.1 (9.4–25.0)	17.2 (8.7–28.5)	0.747
Percentage of the population between 18 and 24 years of age that are less than a high school graduate	14 (7.4–24.5)	14 (7.3–24.7)	13.2 (7.3–21.7)	0.976
Percentage of the population 25 and over with <9th grade educational attainment	6.2 (2.8–13.3)	6.5 (3.1–14.7)	5 (2.0–11.0)	0.306
Percentage of the population 25 years and over that were enrolled in high school but never received a diploma	10.4 (4.8–15.3)	10.3 (5.1–14.8)	10.6 (3.5–16.1)	0.573
Percentage of the occupied housing units with 1.51 or more occupants per room	0 (0.0–1.5)	0 (0.0–1.4)	0.2 (0.0–1.7)	0.386
Unemployment rate for population 16 years and over	7.4 (5.1–14.4)	7.4 (5.2–15.5)	7.2 (5.0–10.3)	0.826
Percentage of the households with a male head of the household with no spouse present	6.5 (3.5–10.5)	7 (3.9–10.7)	4.95 (2.5–9.8)	0.172

^a^IQR (25th percentile, 75th percentile).

### ICU admission

Table 4 reports the adjusted ORs for inpatient ICU admission after adjusting for covariates, comorbidities, and neighborhood characteristics. With regard to insurance, patients who had public insurance or who were uninsured had lower odds of ICU admission (OR, 0.24; 95% CI, 0.09–0.63; *P* = 0.004) compared with those with private insurance. Neighborhood factors were not statistically significantly associated with ICU admission. Race and ethnicity were not included in the bivariate and GEE analyses for ICU admission because of low aggregate counts.

**Table 4.** GEE: associations between inpatient ICU use in relation to independent variables, *n* = 124.

	Model 1: socioeconomic characteristics		Model 2: addition of comorbidities		Model 3: addition of neighborhood characteristics	
Variable^a^	Adjusted OR (95% CI)	*P*-value	Adjusted OR (95% CI)	*P*-value	Adjusted OR (95% CI)	*P*-value
*Inpatient ICU use*						
Sex: female	0.34 (0.13–0.91)	0.032	0.35 (0.13–0.97)	0.043	0.33 (0.11–0.96)	0.043
Payer: public/uninsured	0.24 (0.09–0.63)	0.004	0.26 (0.10–0.68)	0.006	0.24 (0.08–0.66)	0.006
Age	1.25 (1.09–1.45)	0.002	1.26 (1.10–1.48)	0.004	1.26 (1.05–1.52)	0.012
Date of visit: wave 3	0.22 (0.09–0.55)	0.001	0.30 (0.12–0.77)	0.012	0.35 (0.14–0.90)	0.030
Comorbidities: 2 or more			3.74 (1.47–9.53)	0.006	3.84 (1.41–10.45)	0.008
Percentage of the households below the federal poverty line in the past 12 months					0.97 (0.91–1.04)	0.381
Percentage of the population between 18 and 24 years of age that are less than a high school graduate					1.01 (0.98–1.05)	0.52
Percentage of the population 25 and over with <9th grade educational attainment					0.97 (0.86–1.09)	0.567
Percentage of the population 25 years and over that were enrolled in high school but never received a diploma					1.04 (0.90–1.20)	0.601
Percentage of the occupied housing units with 1.51 occupants or more per room					0.87 (0.60–1.27)	0.480
Unemployment rate for population 16 years and over					1.02 (0.93–1.11)	0.729
Percentage of the households with a male being the head of the household with no spouse present					1.07 (0.92–1.24)	0.404
ROC curve area	0.80		0.84		0.86	

^a^Reference groups: male, non-Hispanic Black/other, private, waves 1 and 2, Rush Copley Medical Center and Oak Park Hospital, 0–1 comorbidities.

### Inpatient oxygen use demographic characteristics

Of the 124 inpatient admissions, 58.1% required oxygen assistance (*n* = 72; Table 5). The median age of patients needing oxygen assistance was 21 years (IQR = 19–23). Public insurance or uninsured was most common (69.4%) among inpatients requiring oxygen assistance, followed by private insurance (30.6%). More than half of the patients requiring oxygen assistance were females (62.5%) and >70% of patients on oxygen assistance were seen at RUMC (*n* = 52). Over 50% of patients admitted to the hospital requiring oxygen assistance were seen in waves 1 and 2 (*n* = 38) and more patients with 0 or 1 comorbidity required oxygen assistance (*n* = 39) compared with those with 2 or more comorbidities (*n* = 33). Chi-square analyses in Table 5 reveal that date of visit (*P* = 0.002), hospital visited (*P* < 0.001), and comorbidities (*P* = 0.055) were significant toward oxygen utilization for inpatient admissions, while neighborhood factors were not.

**Table 5.** Description of the sample of patients by oxygen use status, *n* = 124.

	Total *n* = 124	No oxygen *n* = 52 (41.9%)	Oxygen use *n* = 72 (58.1%)	*P*-value
Sex, *n* (%)				0.437
Male	43 (34.7)	16 (30.8)	27 (37.5)	
Female	81 (65.3)	36 (69.2)	45 (62.5)	
Payer, *n* (%)				0.498
Private	35 (28.2)	13 (25.0)	22 (30.6)	
Public/uninsured	89 (71.8)	39 (75.0)	50 (69.4)	
Age in years, median (IQR^a^)	21 (18–23)	20.5 (17–22)	21 (19–23)	0.153
Date of visit				0.002
Waves 1 and 2	51 (41.1)	13 (25.0)	38 (52.8)	
Wave 3	73 (58.9)	39 (75.0)	34 (47.2)	
Hospital, *n* (%)				<0.001
RUMC	73 (58.9)	21 (40.4)	52 (72.3)	
Rush Copley Medical Center and Oak Park Hospital	51(41.1)	31(59.6)	20 (41.7)	
Comorbidities, *n* (%)				0.055
0–1 comorbidities	76 (61.3)	37 (71.2)	39 (54.2)	
2 or more comorbidities	48 (38.7)	15 (28.8)	33 (45.8)	
Neighborhood characteristics, median (IQR^a^)				
Percentage of the households below the federal poverty line in the past 12 months	16.5 (9.3–25.3)	19.9 (10.6–27.0)	15.3 (8.5–24.6)	0.302
Percentage of the population between 18 and 24 years of age that are less than a high school graduate	14 (7.4–24.5)	16.2 (10.9–24.8)	11.8 (6.9–24.1)	0.112
Percentage of the population 25 and over with <9th grade educational attainment	6.2 (2.8–13.3)	5.3 (5.3–13)	6.6 (3–14.6)	0.399
Percentage of the population 25 years and over that were enrolled in high school but never received a diploma	10.4 (4.8–15.3)	11.2 (4.9–15.9)	10.3 (4.4–14.1)	0.353
Percentage of the occupied housing units with 1.51 or more occupants per room	0 (0.0–1.5)	0 (0.0–1.3)	0 (0.0–1.5)	0.703
Unemployment rate for population 16 years and over	7.4 (5.1–14.4)	7.5 (5.1–14.1)	7.4 (5.1–14.9)	0.773
Percentage of the households with a male head of the household with no spouse present	6.5 (3.5–10.5)	6.2 (3–10.7)	6.45 (3.6–10.2)	0.853

^a^IQR (25th percentile, 75th percentile).

### Inpatient oxygen utilization

Table 6 demonstrates adjusted ORs for inpatient oxygen use after adjusting for covariates, comorbidities, and neighborhood characteristics. Insurance/payer status and neighborhood characteristics displayed no significance in relation to oxygen administered through ventilation, intubation, and through additional oxygen assistance mechanisms for inpatients. Race and ethnicity were not included in bivariate and GEE analyses for oxygen use because of low aggregate counts, resulting in potential patient identity exposure and a bad model fit.

**Table 6.** GEE: associations between inpatient oxygen use in relation to independent variables, *n* = 124.

	Model 1: socioeconomic characteristics		Model 2: addition of comorbidities		Model 3: addition of neighborhood characteristics	
Variable^a^	Adjusted OR (95% CI)	*P*-value	Adjusted OR (95% CI)	*P*-value	Adjusted OR (95% CI)	*P*-value
*Inpatient oxygen use*						
Sex: female	0.75 (0.32–1.77)	0.510	0.76 (0.319–1.80)	0.531	0.70 (0.28–1.73)	0.437
Payer: public/uninsured	0.95 (0.38–2.35)	0.911	0.96 (0.38–2.38)	0.922	0.89 (0.35–2.31)	0.815
Age	0.85 (0.74–0.98)	0.025	0.85 (0.74–0.98)	0.026	0.86 (0.74–1.00)	0.047
Date of visit: wave 3	0.45 (0.19–1.03)	0.058	0.46 (0.20–1.08)	0.074	0.41 (0.17–0.99)	0.047
Hospital: RUMC	3.80 (1.64–8.82)	0.002	3.66 (1.56–8.61)	0.003	3.74 (1.54–9.10)	0.004
Comorbidities: 2 or more			1.20 (0.53–2.72)	0.660	1.10 (0.50–2.50)	0.863
Percentage of the households below the federal poverty line in the past 12 months					1.02 (0.96–1.08)	0.551
Percentage of the population between 18 and 24 years of age that are less than a high school graduate					1.03 (0.99–1.06)	0.194
Percentage of the population 25 and over with <9th grade educational attainment					0.98 (0.91–1.05)	0.507
Percentage of the population 25 years and over that were enrolled in high school but never received a diploma					1.01 (0.90–1.12)	0.911
Percentage of the occupied housing units with 1.51 occupants or more per room					0.99 (0.72–1.40)	0.952
Unemployment rate for population 16 years and over					0.98 (0.91–1.06)	0.644
Percentage of the households with a male being the head of the household with no spouse present					0.97 (0.84–1.11)	0.637
ROC curve area	0.74		0.74		0.75	

^a^Reference groups: male, non-Hispanic Black/other, private, waves 1 and 2, Rush Copley Medical Center and Oak Park Hospital, 0–1 comorbidities.

## Discussion

This retrospective analysis reviewed patient data for 1,057 individuals in an urban Midwest health system, with the purpose of determining whether disparities exist in COVID-19 outcomes for adolescents and young adults. Our data suggest that racial and ethnic minority status was associated with hospital admission, while insurance/payer status was related to ICU admission. Non-Hispanic White youth (OR, 2.96; 95% CI, 1.61–5.46; *P* = 0.001) and Hispanic youth (OR, 3.34; 95% CI, 1.84–6.10; *P* < 0.001) had higher odds of hospital admission compared with non-Hispanic Black/other youth after adjusting for sex, age, encounter hospital, and date of visit. Source of insurance was not included into the GEE models for hospital use because of low frequencies in outcome categories, which may produce unreliable model effect estimates and result in a bad model fit.

Patients with public insurance or those who were uninsured were less likely to be admitted to the ICU (OR, 0.24; 95% CI, 0.09–0.64; *P* = 0.004) compared with those with private insurance after adjusting for socioeconomic characteristics. Patients who presented with 2 or more comorbidities were more likely to be admitted to the ICU (OR, 3.74; 95% CI, 1.46–9.59; *P* = 0.006) and require oxygen assistance (OR, 1.20; 95% CI, 0.51–2.84; *P* = 0.675) compared with those with one or fewer comorbidities. Assessing hospital utilization and presentation severity among vulnerable youth and young adults is essential, because the identification of patterns related to COVID-19 health outcomes can be used for creating new public policies and health-related services to reduce such disparities within this subpopulation.

Based on prior findings addressing race and ethnicity and adult COVID-19 outcomes (16–18, 44), we hypothesized that Hispanic and non-Hispanic Black/other adolescents and young adults were more likely to be hospitalized compared with non-Hispanic White youth and young adults. However, we found that non-Hispanic White youth were nearly three times as likely to be hospitalized due to COVID-19 compared with non-Hispanic Black/other youth. Data regarding the reasons underlying why many underserved individuals present to the ED but are not admitted to the hospital are limited, yet it is important to remain cognizant of numerous causes that could promote differences in hospitalization rates for racial and ethnic minority individuals compared with non-Hispanic White individuals. For example, one study demonstrated that pulse oximetry measurements depicted a persistent overestimation of oxygen saturation in non-White populations (45). Such false elevation readings could impact admission to the hospital and/or ICU for racial and ethnic minority groups. Another study mentioned that factors such as equal access to proper diagnostic testing and care, similar severity of illness at the time of seeking care, attaining better-quality insurance among racial and ethnic groups, improved access to primary care reducing severity of illness for racial and ethnic groups, refusal of hospital admission due to a mistrust of the healthcare system, and presence of provider bias at the time of care could potentially contribute to comparable odds in hospitalizations for racial and ethnic minority individuals compared with non-Hispanic White individuals (38). Other studies have concluded that although race and ethnicity are crucial factors in determining the probability of testing positive for COVID-19, they made less of an impact with regard to severity of illness (16, 38). Although race and ethnicity were included in our GEE models, stratified analyses were performed on race and ethnicity to further understand racial differences in hospitalizations (Tables S1–S3). Stratified analyses are presented; however, because of the smaller sample sizes within the non-Hispanic White strata, the generalizability of results from those models may be limited.

Although prior studies have demonstrated that adults residing in disadvantaged neighborhoods with lower median household incomes, lower educational attainment, poorer vehicle access, higher unemployment rates, and more food insecurity were associated with poorer COVID-19 outcomes such as increased incidence and mortality (20, 21, 30, 46, 47), our findings suggested that neighborhood characteristics investigated at the census tract level were not significantly associated with inpatient admission, ICU admission, or oxygen use for youth and young adults. After dissecting the geographic locations of the youth and young adults investigated in this study, increased density was seen around each hospital within RUSH (RUMC, Rush Copley Medical Center, and Rush Oak Park Hospital). Although infection clusters were present, the range of values for each neighborhood characteristic appeared heterogeneous. Therefore, perhaps utilizing different characterization methods of neighborhoods such as other proxies emerging to assess SDOH association with health outcomes may be more likely to provide macro-level impacts of such neighborhood factors on youth and young adult COVID-19 health outcomes and disparities.

### Limitations and future work

We deem our findings robust and beneficial, as they shed light on the complex relationships between sociodemographic characteristics and COVID-19 health outcomes for vulnerable adolescents and youth. Yet, it is important to recognize several limitations within the present study.

Our sample size was relatively small, because although our hospital admission model utilized a sample size of over 600, our models for ICU admission and oxygen assistance did not use a large sample size and therefore were underpowered. Also, each GEE model investigated a different set of variables to ensure that no variable in the model had a count of <11 to maintain patient confidentiality and to prevent low frequencies in outcome subcategories, which could produce unreliable model effect estimates and result in a bad model fit. Future research could include increasing the sample size of the study to help promote a more well-rounded list of risk factors associated with disease severity.

Our analysis may also have been limited by the small number of non-Hispanic White youth who presented to RUSH, comprising <10% of youth with COVID-19 seen within our hospital system. Although we controlled for the presence of comorbidities within this subgroup, there may have been unobserved heterogeneity across non-Hispanic White youth patients. These youth may have had better access to outpatient care, and as such, presented to the ED with more severe COVID-19 after referral from another provider. However, further research is needed to examine whether the patterns of care prior to the index ED visit differed by race and ethnicity. Consequently, it is important to acknowledge that there were many low-income and racial and ethnic minority patients who presented to the ED but were not admitted to the hospital. We did not have the necessary data to assess whether racial and ethnic minority patients presented with decreased COVID-19 severity outcomes that lead to such decreased hospital admission. Therefore, future research should examine whether lower-income and racial and ethnic minority patients were more likely to be readmitted to the hospital later and also examine longer-term outcomes to understand this decrease in hospital admission within our data set.

Differences in school policies with regard to COVID-19 such as in-person learning vs. online and mandated masking were not accounted for in our analysis. This could play an important role in COVID-19 exposure and disease severity for adolescents and youth. For example, one study mentioned that an increase in COVID-19 cases was directly correlated to the timing of opening for K-12 schools and colleges in the United States, because schools that implemented in-person learning saw a subsequent increase in case and death grow rates (47). Furthermore, the study stated that schools that did not mandate staff to wear masks saw COVID-19 case growth compared with those that promoted masking (47).

Another limitation present in our study is that our data included the first year of COVID-19 in which diagnosis and treatment mechanisms were quickly evolving as the understanding of the disease increased. In addition, our data were limited to youth patients seen at a single academic health system, and the findings may not be generalizable to youth seen within other systems. Thus, addressing the same research problem but outside of the single, metropolitan health system investigated in this study could also help ensure that the study findings are more generalizable and relatable to youth from other towns and cities.

The findings of this retrospective analysis lay the groundwork for a proper understanding of vulnerabilities adolescents and youth experience with regard to health outcomes and healthcare utilization, leading to potential policy creation or change. Although preventive measures are crucial, recovery measures to restore communities of all ages that have experienced the effect of a pandemic in varying ways can also prove to be essential. This could include creating pointed policies for vulnerable youth populations such as homeless youth, immigrant youth, youth of color, and those residing in low-income households. Also, increasing inclusivity by encouraging the involvement of individuals from all ages in rulemaking at the state level can help ensure that the concerns of different age groups are considered in the decision-making process. Finally, understanding the allocation of public resources among different age groups is also essential, because the use of assessments to monitor such distributions can help guarantee that the needs of all age cohorts are met appropriately.

## Supplementary Material

pgad396_Supplementary_Data

## Data Availability

The data utilized in this study are confidential patient EMR data and therefore cannot be shared.
