# Supplementary material for: Racial, socioeconomic, and neighborhood characteristics in relation to COVID-19 severity of illness for adolescents and young adults
Source: PNAS Nexus. 2023 Nov 17;2(11):pgad396. doi: 10.1093/pnasnexus/pgad396 (PMC10682970; doi:10.1093/pnasnexus/pgad396)
Supplement: pgad396_Supplementary_Data [file pgad396_supplementary_data.docx]

| Supplemental Table 1. Stratified Analyses for non-Hispanic White: Associations Between ED and Hospital Admissions in Relation to Independent Variables, n=93. | | | | | | |
| --- | --- | --- | --- | --- | --- | --- |
|  | **Model 1: Socioeconomic Characteristics** | | **Model 2: Addition of Comorbidities** | | **Model 3: Addition of Neighborhood Characteristics** | |
| **Variable*** | Adjusted OR (95%CI) | p-Value | Adjusted OR (95%CI) | p-Value | Adjusted OR (95%CI) | p-Value |
| **ED and Inpatient Admissions** | | | | | | |
| Sex: Female | 1.29 (0.40-4.16) | 0.675 | 2.66 (0.60-11.73) | 0.196 | 3.90 (0.94-16.16) | 0.060 |
| Age | 1.00 (0.85-1.18) | 0.975 | 0.98 (0.82-1.17) | 0.823 | 0.91 (0.74-1.13) | 0.406 |
| Date of Visit: Wave 3 | 1.61 (0.45-5.81) | 0.468 | 2.65 (0.59-11.83) | 0.203 | 7.53 (1.56-36.38) | 0.012 |
| Hospital: Rush University Medical Center | 11.7 (3.43-39.8) | <0.001 | 7.27 (1.93-27.4) | 0.003 | 9.41 (2.03-43.62) | 0.004 |
| Comorbidities: 2 or more |  | | 32.12 (3.13-330.2) | 0.004 | 97.84 (5.63-1700.4) | 0.002 |
| Percent of households below the federal poverty line in the past 12 month |  | |  | | 1.04 (0.96-1.13) | 0.339 |
| Percent of the population between 18-24 years of age that are less than a high school graduate |  | |  | | 0.91 (0.81-1.02) | 0.098 |
| Percent of the population 25 and over with less than 9th grade educational attainment |  | |  | | 1.12 (0.93-1.35) | 0.224 |
| Percent of the population 25 years and over that were enrolled in high school but never received a diploma |  | |  | | 1.06 (0.86-1.30) | 0.590 |
| Percent of occupied housing units with 1.51 occupants or more per room |  | |  | | 0.70 (0.38-1.30) | 0.259 |
| Unemployment rate for population 16 years and over |  | |  | | 0.71 (0.49-1.04) | 0.080 |
| Percent of households with a male being the head of the household with no spouse present |  | |  | | 1.09 (0.87-1.38) | 0.454 |
| *Reference Groups: Male, Non-Hispanic Black/Other, Private, Wave 1 and 2, Rush Copley Medical Center and Oak Park Hospital, 0-1 Comorbidities | | | | | | |

| Supplemental Table 2. Stratified Analyses for Hispanic: Associations Between ED and Hospital Admissions in Relation to Independent Variables, n=443. | | | | | | |
| --- | --- | --- | --- | --- | --- | --- |
|  | **Model 1: Socioeconomic Characteristics** | | **Model 2: Addition of Comorbidities** | | **Model 3: Addition of Neighborhood Characteristics** | |
| **Variable*** | Adjusted OR (95%CI) | p-Value | Adjusted OR (95%CI) | p-Value | Adjusted OR (95%CI) | p-Value |
| **ED and Inpatient Admissions** | | | | | | |
| Sex: Female | 1.45 (0.76-2.75) | 0.260 | 1.64 (0.80-3.36) | 0.177 | 1.66 (0.81-3.41) | 0.169 |
| Age | 0.91 (0.82-1.01) | 0.084 | 10.92 (0.83-1.03) | 0.145 | 0.91 (0.82-1.01) | 0.068 |
| Date of Visit: Wave 3 | 1.59 (0.79-3.18) | 0.191 | 1.32 (0.63-2.77) | 0.463 | 1.36 (0.64-2.90) | 0.421 |
| Hospital: Rush University Medical Center | 2.03 (1.05-3.94) | 0.035 | 1.96 (1.01-3.81) | 0.046 | 1.81(0.83-3.94) | 0.135 |
| Comorbidities: 2 or more |  | | 5.08 (2.27-11.38) | <0.001 | 4.78 (2.15-10.67) | <0.001 |
| Percent of households below the federal poverty line in the past 12 month |  | |  | | 0.98 (0.93-1.04) | 0.478 |
| Percent of the population between 18-24 years of age that are less than a high school graduate |  | |  | | 0.99 (0.96-1.03) | 0.709 |
| Percent of the population 25 and over with less than 9th grade educational attainment |  | |  | | 1.01 (0.97-1.05) | 0.745 |
| Percent of the population 25 years and over that were enrolled in high school but never received a diploma |  | |  | | 1.04 (0.95-1.14) | 0.406 |
| Percent of occupied housing units with 1.51 occupants or more per room |  | |  | | 0.82 (0.67-1.01) | 0.063 |
| Unemployment rate for population 16 years and over |  | |  | | 1.02 (0.96-1.08) | 0.610 |
| Percent of households with a male being the head of the household with no spouse present |  | |  | | 0.96 (0.87-1.06) | 0.447 |
| *Reference Groups: Male, Non-Hispanic Black/Other, Private, Wave 1 and 2, Rush Copley Medical Center and Oak Park Hospital, 0-1 Comorbidities | | | | | | |

| Supplemental Table 3. Stratified Analyses for non-Hispanic Black/Other: Associations Between ED and Hospital Admissions in Relation to Independent Variables, n=521. | | | | | | |
| --- | --- | --- | --- | --- | --- | --- |
|  | **Model 1: Socioeconomic Characteristics** | | **Model 2: Addition of Comorbidities** | | **Model 3: Addition of Neighborhood Characteristics** | |
| **Variable*** | Adjusted OR (95%CI) | p-Value | Adjusted OR (95%CI) | p-Value | Adjusted OR (95%CI) | p-Value |
| **ED and Inpatient Admissions** | | | | | | |
| Sex: Female | 1.62 (0.90-2.89) | 0.106 | 1.95 (1.04-3.68) | 0.038 | 1.91 (1.01-3.61) | 0.046 |
| Age | 1.02 (0.92-1.11) | 0.76 | 1.06 (0.96-1.17) | 0.272 | 1.06 (0.95-1.17) | 0.288 |
| Date of Visit: Wave 3 | 1.90 (1.10-3.26) | 0.021 | 1.95 (1.23-3.37) | 0.016 | 1.95 (1.16-3.28) | 0.012 |
| Hospital: Rush University Medical Center | 1.26 (0.72-2.21) | 0.422 | 1.02 (0.59-1.76) | 0.958 | 1.04 (0.59-1.84) | 0.884 |
| Comorbidities: 2 or more |  | | 7.39 (3.94-13.85) | <0.001 | 7.92 (4.14-15.14) | <0.001 |
| Percent of households below the federal poverty line in the past 12 month |  | |  | | 1.00 (0.96-1.03) | 0.792 |
| Percent of the population between 18-24 years of age that are less than a high school graduate |  | |  | | 1.00 (0.98-1.02) | 0.947 |
| Percent of the population 25 and over with less than 9th grade educational attainment |  | |  | | 1.00 (0.94-1.07) | 0.948 |
| Percent of the population 25 years and over that were enrolled in high school but never received a diploma |  | |  | | 1.02 (0.96-1.09) | 0.460 |
| Percent of occupied housing units with 1.51 occupants or more per room |  | |  | | 1.22 (0.95-1.56) | 0.119 |
| Unemployment rate for population 16 years and over |  | |  | | 0.99 (0.95-1.04) | 0.776 |
| Percent of households with a male being the head of the household with no spouse present |  | |  | | 1.01 (0.92-1.10) | 0.908 |
| *Reference Groups: Male, Non-Hispanic Black/Other, Private, Wave 1 and 2, Rush Copley Medical Center and Oak Park Hospital, 0-1 Comorbidities | | | | | | |

| Supplemental Table 4: Insurance Status Crosstabulations | | | | | |
| --- | --- | --- | --- | --- | --- |
|  | | | | | |
|  |  | **Insurance Status** | |  |  |
|  |  | *Private* | *Public/Uninsured* | Total | P-Value |
| **Comorbidities** | *Less than 2* | 249 (85.6%) | 678 (88.5%) | 927 (87.7%) | 0.193 |
|  | *2 or more* | 42 (14.4%) | 88 (11.5%) | 130 (12.3%) |  |
| Total |  | 291 | 766 | 1057 |  |
|  |  |  |  |  |  |
| **Date of Visit** | *Wave 1 & 2* | 157 (54.0%) | 396 (51.7%) | 553 (52.3%) | 0.512 |
|  | *Wave 3* | 134 (46.05) | 370 (48.3%) | 504 (47.7%) |  |
| Total |  | 291 | 766 | 1057 |  |
|  |  |  |  |  |  |
| **Hospital Visited** | *Rush University Medical Center* | 125 (43.0%) | 354 (46.2%) | 479 (45.3%) | 0.342 |
|  | *Rush Copley Medical Center and Oak Park Hospital* | 166 (57.0%) | 412 (53.8%) | 578 (54.7%) |  |
| Total |  | 291 | 766 | 1057 |  |
|  |  |  |  |  |  |
| **ICU Admission** | *No* | 275 (94.5%) | 744 (97.1%) | 1019 (96.4%) | 0.041 |
|  | *Yes* | 16 (5.5) | 22 (2.9%) | 38 (3.6%) |  |
| Total |  | 291 | 766 | 1057 |  |
|  |  |  |  |  |  |
| **Oxygen Use** | *No Oxygen* | 263 (90.4%) | 711 (92.8%) | 974 (92.1%) | 0.187 |
|  | *Oxygen* | 28 (9.6%) | 55 (7.2%) | 83 (7.9%) |  |
| Total |  | 291 | 766 | 1057 |  |
|  |  |  |  |  |  |
| **Sex** | *Male* | 133 (45.7%) | 316 (41.3%) | 449 (42.5%) | 0.191 |
|  | *Female* | 158 (54.3%) | 450 (58.7%) | 608 (57.5%) |  |
| **Total** |  | 291 | 766 | 1057 |  |
|  |  |  |  |  |  |
| **Race and Ethnicity** | *Non-Hispanic White* | 58 (19.9%) | 35 (4.6%) | 93 (8.8%) | <0.001 |
|  | *Hispanic* | 125 (43.0%) | 318 (41.5%) | 443 (41.9%) |  |
|  | *Non-Hispanic Black and Other* | 108 (37.1%) | 413 (53.9% | 521 (49.3% |  |
| **Total** |  | 291 | 766 | 1057 |  |

| Supplemental Table 5: Race and Ethnicity Crosstabulations | | | | | | |
| --- | --- | --- | --- | --- | --- | --- |
|  | | | | | | |
|  |  | **Race and Ethnicity** | | |  |  |
|  |  | *Non-Hispanic White* | *Hispanic* | Non-Hispanic Black and Other | Total | P-Value |
| **Comorbidities** | *Less than 2* | 83 (89.2%) | 399 (90.1%) | 445 (85.4%) | 927 (87.7%) | 0.081 |
|  | *2 or more* | 10 (10.8%) | 44 (9.9%) | 76 (14.6%) | 130 (12.3%) |  |
| Total |  | 93 | 443 | 521 | 1057 |  |
|  |  |  |  |  |  |  |
| **Date of Visit** | *Wave 1 & 2* | 38 (40.9%) | 272 (61.4%) | 243 (46.6%) | 553 (52.3%) | <0.001 |
|  | *Wave 3* | 55 (59.1%) | 171 (38.6%) | 278 (53.4%) | 504 (47.7%) |  |
| Total |  | 93 | 443 | 521 | 1057 |  |
|  |  |  |  |  |  |  |
| **Hospital Visited** | *Rush University Medical Center* | 29 (31.2%) | 187 (42.2%) | 263 (50.5%) | 479 (45.3%) | <0.001 |
|  | *Rush Copley Medical Center and Oak Park Hospital* | 64 (68.8%) | 256 (57.8%) | 258 (49.5%) | 578 (54.7%) |  |
| Total |  | 93 | 443 | 521 | 1057 |  |
|  |  |  |  |  |  |  |
| **ICU Admission** | *No* | 83 (89.2%) | 432 (97.5%) | 504 (96.7%) | 1019 (96.4%) | <0.001 |
|  | *Yes* | 10 (10.8%) | 11 (2.5%) | 17 (3.3%) | 38 (3.6%) |  |
| Total |  | 93 | 443 | 521 | 1057 |  |
|  |  |  |  |  |  |  |
| **Oxygen Use** | *No Oxygen* | 75 (80.6%) | 413 (93.2%) | 486 (93.3%) | 974 (92.1%) | <0.001 |
|  | *Oxygen* | 18 (19.4%) | 30 (6.8%) | 35 (6.7%) | 83 (7.9%) |  |
| Total |  | 93 | 443 | 521 | 1057 |  |
|  |  |  |  |  |  |  |
| **Sex** | *Male* | *38 (40.9%)* | 194 (43.8%) | 217 (41.7%) | 449 (42.5%) | 0.756 |
|  | *Female* | 55 (59.1%) | 249 (56.2%) | 304 (58.3%) | 608 (57.5%) |  |
| Total |  | 93 | 443 | 521 | 1057 |  |
|  |  |  |  |  |  |  |
| I**nsurance Status** | *Private* | 58 (19.9%) | 125 (43.0%) | 108 (37.1%) | 291 (27.5%) | <0.001 |
|  | *Public/Uninsured* | 35 (4.6%) | 318 (41.5%) | 413 (53.9%) | 766 (72.5%) |  |
| Total |  | 93 | 443 | 521 | 1057 |  |
|  |  |  |  |  |  |  |
